# Booster dose of BNT162b2 after two doses of CoronaVac improves neutralization of SARS-CoV-2 Omicron variant

**DOI:** 10.1038/s43856-022-00141-4

**Published:** 2022-06-29

**Authors:** Guilherme R. F. Campos, Nathalie Bonatti Franco Almeida, Priscilla Soares Filgueiras, Camila Amormino Corsini, Sarah Vieira Contin Gomes, Daniel Alvim Pena de Miranda, Jéssica Vieira de Assis, Thaís Bárbara de Souza Silva, Pedro Augusto Alves, Gabriel da Rocha Fernandes, Jaquelline Germano de Oliveira, Paula Rahal, Rafaella Fortini Queiroz Grenfell, Maurício L. Nogueira

**Affiliations:** 1 Laboratório de Pesquisas em Virologia (LPV), Faculdade de Medicina de São José do Rio Preto (FAMERP), São José do Rio Preto, Brazil; 2 Diagnosis and Therapy of Infectious Diseases and Cancer, Oswaldo Cruz Foundation (Fiocruz), Belo Horizonte, Brazil; 3 Laboratório de Imunologia de Doenças Virais, Instituto Rene Rachou—Fundação Oswaldo Cruz, Belo Horizonte, Brazil; 4 Laboratório de Imunologia Celular e Molecular, Instituto Rene Rachou—Fundação Oswaldo Cruz, Belo Horizonte, Brazil; 5 Laboratório de Estudos Genômicos, Departamento de Biologia, Instituto de Biociências Letras e Ciências Exatas (IBILCE), Universidade Estadual Paulista (Unesp), São José do Rio Preto, Brazil; 6 Department of Infectious Diseases, College of Veterinary Medicine, University of Georgia, Athens, GA USA; 7 Hospital de Base, São José do Rio Preto, Brazil; 8 Department of Pathology, University of Texas Medical Branch, Galveston, TX USA

**Keywords:** Virology, Viral infection

## Abstract

**Background:**

The emergence of the new SARS-CoV-2 Omicron variant, which is known to have a large number of mutations when compared to other variants, brought to light the concern about vaccine escape, especially from the neutralization by antibodies induced by vaccination.

**Methods:**

Based on viral microneutralization assays, we evaluated in 90 individuals the impact on antibody neutralization induction, against Omicron variant, by a booster dose of BNT162b2 mRNA vaccine after the CoronaVac primary vaccination scheme.

**Results:**

Here we show that the percentage of seroconverted individuals 30 and 60 days after CoronaVac scheme was 16.6% and 10%, respectively. After booster dose administration, the seroconvertion rate increased to 76.6%. The neutralization mean titer against Omicron in the CoronaVac protocol decreased over time, but after the booster dose, the mean titer increased 43.1 times.

**Conclusions:**

These results indicate a positive impact of this vaccine combination in the serological immune response against SARS-CoV-2 Omicron variant.

## Plain language summary

The high transmission rates of SARS-CoV-2 favor the appearance of variants of the virus bearing a high number of changes in the genetic material, such as the Omicron variant. These changes might impact the efficacy of COVID-19 vaccines. Here, we evaluated the ability, of a highly adopted vaccination scheme in Brazil, of producing neutralizing antibodies, proteins present in the human body that bind to the virus and prevent infection, against the Omicron variant. This scheme consisted in two doses of the CoronaVac vaccine followed by an additional dose of the BNT162b2 vaccine. Our analysis showed that, before the additional dose, the number of individuals presenting neutralizing antibodies against Omicron was low and decreased over time. However, the production of neutralizing antibodies increased significantly after the BNT162b2 dose. Our findings support that this vaccination scheme improves the neutralization of Omicron.

## Abstract (web summary)

Campos et al. assessed the impact of a booster dose of BNT162b2 on antibody neutralization against the SARS-CoV-2 Omicron variant, following two doses of the CoronaVac vaccine. Neutralization mean titers against Omicron decrease over time following two doses of CoronaVac but increase again after the BNT162b2 booster, as does seroconversion.

## Introduction

The vaccines against COVID-19 presented an important impact reducing the number of cases, hospitalization and deaths worldwide. However, in some countries the access to vaccination is far from the ideal, where the spread of SARS-CoV-2 continues at high rates favoring the emergence of variants. Indeed, since the beginning of the pandemics, the world has faced a continuous occurrence of variants, which is a natural process for a highly transmissible virus like SARS-CoV-2. Among these variants, the increase of transmission rate, augmented severity of cases and possibility to escape from immune response, stimulated by vaccination or previous infection, are the main concerns surrounding the called variants of concern (VOCs)^1,2^. The Omicron variant (B.1.1.529) has called attention due to its high number of mutations across the whole genome when compared to other VOCs. The Omicron variant accumulates at least 47 mutations in its whole genome (the highest number among variants), of which more than half are present in the Spike protein, the main target of the serological immune response induced by the majority of COVID-19 vaccines^3–5^.

In a global panorama, Brazil stands out as one of the epicenters for viral dissemination, presenting high numbers of cases and deaths. Along with USA and India, Brazil ranks in the top three of the most impacted countries, with more than 25 million cases and 625,000 deaths until February 7, 2022^6^. On the other hand, historically, Brazil has a solid immunization program, which leaded to a huge SARS-CoV-2 vaccination adherence when compared to other countries, reaching more than 352 million doses administrated in the population, and almost 152 million people fully vaccinated. Among the distributed vaccines in Brazil, CoronaVac, the most used vaccine worldwide, was approved by our regulatory agency in January of 2021. This vaccine uses an inactivated virus technology and was the most administrated vaccine in Brazil until the middle of 2021, inoculated especially in elderly and healthcare workers individuals^7,8^. The scheme consisted in a two doses protocol, with 2–4 weeks between doses. It is estimated that, until January of 2022, around 85 million doses of CoronaVac were administrated in the Brazilian population. With the introduction of some VOCs in the country, especially Delta (B.1.617.2) and Omicron variants, the Brazilian government adopted the distribution of a booster dose of the mRNA BNT162b2 vaccine (BioNTech/Pfizer) to enhance immune protection against COVID-19^9^.

In this context, our study reports the positive impact of the booster dose with BNT162b2 vaccine in a Brazilian cohort, under the CoronaVac two doses scheme, upon the stimulation of neutralizing antibodies against Omicron variant. Here we show that the combination of these vaccines not only improves the number of seroconverted individuals, but also increases the titer of neutralizing antibody in the vaccinated population.

## Methods

### Cohort definition and serum samples collection

To evaluate the presence of neutralizing antibodies induced by vaccination, the selected participants were submitted to a vaccination scheme consisting of the inoculation with two 0.5 ml shots of CoronaVac (600 SU per dose) in the original protocol, receiving a 0.3 ml booster of BNT162b2 (30 µg of spike mRNA per dose). This is a part of a major study evaluating the CoronaVac vaccination protocol that it is published as a preprint at SSRN^10^. A total of 90 individuals, randomly selected, were included in the study. All of them were health care workers from two hospitals in Belo Horizonte (Minas Gerais, Brazil) that received CoronaVac primary vaccination protocol in the beginning of 2021. The cohort general characteristics are summarized in Table 1, while the full detailed data, for each included individual, can be found in Supplementary Table 1. They were divided equally into three different groups (30 clinical samples per group): the D30 group, where serum samples were collected 30 days after CoronaVac second dose; the D60 group, composed by samples collected 60 days after second dose; and D270 group, a time point where samples were collected 270 days after CoronaVac second dose and also after BNT162b2 booster dose (30 days after the mRNA vaccine administration).

**Table 1:** General characteristics of the cohort included in the study.

Age	
18–30	21
31–50	56
51–62	13
Gender	
Male	22
Female	68
Comorbidities^a^	
Present	24
Absent	66
Prior infection	
Yes	11
No	79

^a^Asthma, chronic rhinitis, chronic sinusitis, diabetes, dyslipidemia, hyperthyroidism, hypothyroidism, obesity, systemic arterial hypertension, tobacco smoker.

Ethical approval was given by the Ethical Review Committee (CAAE 2898621.9.0000.5091). The included participants provided their written informed consent to participate in this study.

### Cell culture

To perform the in vitro neutralization assays, Vero (ATCC CCL-81) cells were cultured in Dulbecco’s Modified Eagles Medium (DMEM) supplemented with 10% fetal bovine serum (FBS), 100 U/ml of penicillin and 100 µg/ml of streptomycin in a water-jacked incubator, at 37 °C and 5% CO_2_. According to ATCC control quality, mycoplasma contamination was not detected.

### Viral microneutralization assay

In order to evaluate the antibody neutralization levels against SARS-CoV-2 Omicron variant, in samples collected from a cohort vaccinated with the CoronaVac two doses protocol, followed by the BNT162b2 mRNA vaccine booster dose, a viral microneutralization (VNT) was performed. In a day before, 10^4^ Vero cells were distributed, per well, in clear flat bottom 96-well plates and incubated for 24 h at 37 °C and 5% CO_2_, reaching 90–95% of confluence after incubation. Serum samples, collected from the cohort participants, were heat-inactivated for 30 min, at 56 °C. To analyze the neutralization capacity of antibodies induced by vaccination, using microdilution 96-well plates, the inactivated samples were serially diluted in fresh DMEM media supplemented with 100 U/ml of penicillin, 100 µg/ml of streptomycin and 2% FBS. For each serum sample, eight dilutions were tested (1:20, 1:40, 1:80, 1:160, 1:320, 1:640, 1:1280 e 1:2480). The fresh diluted samples were incubated with the live SARS-CoV-2 Omicron variant (HIAE—W.A) in a constant concentration of 50 TCID_50_/ml, at 37 °C, for 1 h. After this period, the diluted samples, mixed with virus, were distributed in the Vero CCL-81 cell plates and maintained in the humidified incubator at 37 °C and 5% CO_2_ for 72 h. Thereafter, media containing serum and viruses was discarded, cells were fixed with 10% formaldehyde, for 20 min, and stained with crystal violet. The neutralization capacity was determined by the presence or absence of cytopathic effect across the dilutions, and for each sample, it was obtained the reciprocal dilution value where 50% of cytopathic effect was avoided (VNT_50_). All samples, at all dilutions, were tested in triplicate and VNT_50_ was calculated by the use of the Spearman-Karber algorithm^10,11^. To validate the assay, a serum sample already known to neutralize Omicron and a sample collected before the pandemics were used as positive and negative control, respectively.

### Statistics and reproducibility

For each time point evaluated during the viral microneutralization assay, 30 biologically independent serum samples were analyzed. The statistical analysis was obtained by one-way ANOVA and Tukey’s multiple comparisons test, considering statistically significant when *p* value < 0.05.

### Reporting summary

Further information on research design is available in the Nature Research Reporting Summary linked to this article.

## Results

### Antibody neutralization capacity against SARS-CoV-2 Omicron variant is enhanced by BNT162b2 booster dose

**Fig. 1:**
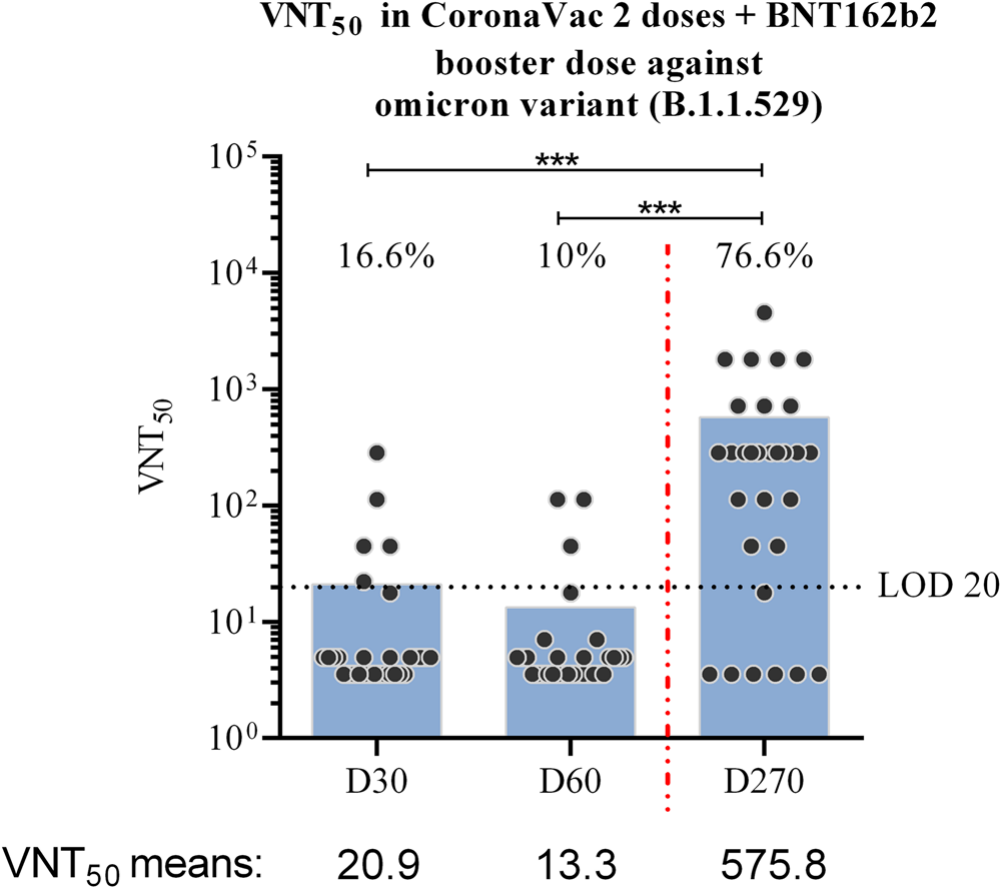
Viral microneutralization assay against SARS-CoV-2 Omicron variant. Neutralizing antibody titers were determined using serum samples collected: 30 days after CoronaVac second dose (D30); 60 days after CoronaVac second dose (D60); 270 days after CoronaVac second dose and 30 days after BNT162b2 booster (D270). For each group, *N* = 30 biologically independent samples. The dark gray circles represent the included participants, and the 50% neutralizing titer (VNT_50_) for each sample was assayed using the Omicron variant live virus (HIAE—W.A). The blue bars represent the VNT_50_ mean values. The black dashed line represents the lower limit of detection (LOD), determined in this assay as 20, while red dashed line represents booster dose time point (D270). The percentage of seroconverted samples, for each group, is indicated in the graph. The statistical analysis was obtained by one-way ANOVA and Tukey’s multiple comparisons test, and the three asterisks represent the *p* value < 0.0001.

Using the viral microneutralization assay (VNT_50_) with SARS-CoV-2 Omicron variant live virus, and analyzing the percentage of seroconvertion, our results showed that after 30 days of second dose (D30), CoronaVac immunization generated detectable neutralizing antibodies against Omicron in only 16.6% of the included individuals. When comparing to the group collected 60 days after second dose (D60), the number of samples presenting neutralizing antibodies dropped to 10%, showing that, as expected, the serological immune response level, that was already low for the Omicron variant, continued to decrease. However, after administration of BNT162b2 booster dose (D270), the number of samples presenting neutralizing antibodies against Omicron significantly increased, reaching almost 76.6% of the included participants (Fig. 1). The evaluation of VNT_50_ mean titers, for each time point, shows that for D270, the mean titer was increased 27.5 times when compared with the mean titer observed for D30. In comparison with D60, this difference increases 43.1 times. These data show that the booster dose with BNT162b2, after CoronaVac vaccination, has improved the production of neutralizing antibodies against the Omicron variant.

## Discussion

In the process of developing vaccines, to achieve an effectiveness level that is acceptable for the regulatory agencies, it is expected that the new vaccine is able to stimulate the immune response in both cellular and serological levels. Thus, the detection and quantification of neutralizing antibodies is one of the fundamental steps during this procedure. In a pandemic situation, some unusual measures are necessary, such as the combination of different vaccines, with different immunological mechanisms, to improve protection, and in these cases the immune response follow-up is crucial. In this scenario, our data showed that BNT162b2 mRNA vaccine booster dose, after a full vaccination protocol with CoronaVac inactivated virus vaccine, is capable to increase the antibody neutralization capacity against SARS-CoV-2 Omicron variant.

The neutralizing improvement here described could be explained by the fact that beta-coronaviruses, including SARS-CoV-2 variants, share a conserved site into Spike protein. Since BNT162b2 vaccine uses Spike mRNA from the original Wuhan SARS-CoV-2 isolate, this conserved region could allow the recognition of Omicron variant by the induced antibodies, independently on the number of accumulated mutations^11^. Furthermore, our findings are in agreement with recent studies that also evaluated antibody neutralization pattern in similar vaccination protocols. The work of Khong et al. showed that, among different vaccine combinations, the use of BNT162b2 after CoronaVac presented higher immunogenicity against SARS-CoV-2, including the Omicron variant^12^, and this is reinforced by our data, where we tested a higher number of samples. Likewise, the study conducted by Cheng et al. highlighted that the combination of BNT162b2 booster dose and CoronaVac two doses scheme achieved 80% of seroconvertion (24 of 30 individuals)^13^, close to the 76.6% observed in our assays (23 of 30).

It is worth to notice that our data corroborate with some findings recently published by GeurtsvanKessel et al. analyzing the BNT162b2 booster dose effect in other vaccination schemes, both in the serological and cellular immune response^14^. In the study, the authors evaluated four different immunization schemes, two using adenovirus-vector technologies and two using the mRNA platform (including BNT162b2 original scheme). They observed that neutralizing antibodies against Omicron variant were significantly lower or absent, depending on the used vaccine, when compared to VOCs like Beta and Delta. However, just as showed by our data, the booster dose with BNT162b2 restored neutralization titers against Omicron for all the tested schemes. It is interesting to highlight that the cellular response against Omicron, mediated by T-cells, was maintained in all the tested vaccination protocols, showing that the highest impact of this variant in the immune response is mainly in the antibody neutralization ability^14^.

From the clinical point of view, a recent article showed, in a Brazilian cohort, that administration of BNT162b2 after CoronaVac increased protection against infection when comparing to CoronaVac original protocol. The CoronaVac two doses scheme presented effectiveness of 34.7% against new infections when compared to unvaccinated individuals, and this protection showed to decrease over time. However, the administration of BNT162b2 booster was responsible to increased protection against infection to 82.6%. The same pattern was observed for COVID-19 disease progression, showing that the booster dose reduces the chances of severe outcomes, especially in risk groups^15^. Another study, also from the clinical area, but analyzing this booster after the BNT162b2 original vaccination scheme, showed a reduction of 90% in the mortality rate^16^. Once our results describe the protection improvement caused by BNT162b2 booster from the perspective of the serological response, all of these studies complement our observations, showing the impact of this immunization approach on distinct branches of the immune system.

As limitations of our study, the neutralization analyses were performed randomly, with different individuals for each time point. Despite bringing heterogeneity to the study and representing a more realistic picture of the real life situation, without selection bias, performing these analyses using samples from the same patients over the time-points, as a follow-up, could bring some different insights about serological response durability. Also, it is well known that serological response is not the only immunological aspect that is stimulated by the vaccination, and that cellular immunity, in association with clinical data, is required to successfully determine the effectiveness of a vaccine. Nevertheless, the data here presented are strengthened by the study of GeurtsvanKessel et al.^14^, emphasizing the need for constant monitoring of neutralizing antibody capacity, especially given the possibility of novel variants emerging in the future.

Thus, our data show that, against Omicron variant, neutralization titers induced by primary vaccination with CoronaVac decreased with time and was 43.1 times lower when compared to the use of a booster dose with BNT162b2. Therefore, this neutralization enhancement after booster dose could reflect, clinically, in higher protection and lower risk of disease progression.

## Supplementary information

Supplementary Information

Supplementary Data

Description of Additional Supplementary Files

Reporting Summary

## Data Availability

The source data that support the findings of this study are available as Supplementary Data.
