# Supplementary material for: Booster dose of BNT162b2 after two doses of CoronaVac improves neutralization of SARS-CoV-2 Omicron variant
Source: Commun Med (Lond). 2022 Jun 29;2:76. doi: 10.1038/s43856-022-00141-4 (PMC9242982; doi:10.1038/s43856-022-00141-4)
Supplement: Supplementary file 1 — Supplementary Information [file 43856_2022_141_MOESM1_ESM.pdf]

SUPPLEMENTARY TABLE 1

ID	Age	Gender	Comorbidities	Prior infection
7	31-50	Male	No	No
9	31-50	Male	No	May 2020
16	31-50	Female	No	June 2020
18	31-50	Female	Hypothyroidism	No
22	18-30	Female	No	No
39	31-50	Male	No	No
47	18-30	Female	No	No
48	31-50	Female	No	No
69	31-50	Male	Systemic arterial hypertension	No
75	31-50	Female	No	No
75	31-50	Female	No	No
98	51-62	Female	No	No
100	51-62	Female	Hypothyroidism - Dyslipidemia - Systemic arterial hypertension	No
111	31-50	Female	No	No
129	31-50	Female	No	December 2020
132	51-62	Male	No	No
151	31-50	Female	No	No
152	18-30	Female	Diabetes	No
156	31-50	Female	No	No
160	31-50	Female	Asthma	No
170	31-50	Female	Systemic arterial hypertension	No
177	18-30	Female	No	No
182	18-30	Male	No	No
186	31-50	Female	Hyperthyroidism	No
199	18-30	Female	No	No
206	51-62	Female	Systemic arterial hypertension - Tobacco smoker	No
224	31-50	Male	No	No
225	31-50	Male	No	December 2020
231	31-50	Female	No	No
241	31-50	Female	No	No
260	18-30	Female	No	No
274	51-62	Female	No	No
302	31-50	Female	No	No
304	31-50	Female	No	No
305	51-62	Female	Diabetes - Hypothyroidism	No
307	18-30	Female	No	No
321	18-30	Female	No	No
324	51-62	Male	No	No
326	18-30	Female	No	No
336	31-50	Female	No	No
341	31-50	Female	No	No
353	31-50	Female	No	No
362	18-30	Female	Tobacco smoker	July 2020
372	51-62	Female	Systemic arterial hypertension	No
375	18-30	Male	No	No
382	31-50	Male	Tobacco smoker	No
386	31-50	Male	Obesity - Diabetes	No
392	51-62	Female	Tobacco smoker	No
394	31-50	Female	Chronic rhinitis - Chronic sinusitis	No
396	31-50	Female	No	No
402	18-30	Female	No	No
421	31-50	Female	No	September 2020
426	51-62	Female	Systemic arterial hypertension	No
438	31-50	Male	Systemic arterial hypertension	August 2020
440	18-30	Female	No	December 2020
441	31-50	Male	No	No
444	51-62	Female	No	No
446	31-50	Female	No	No
448	31-50	Female	No	No
452	18-30	Female	No	No
469	51-62	Female	Systemic arterial hypertension	No
491	31-50	Female	No	No
512	31-50	Female	Hypothyroidism	No
523	31-50	Female	Systemic arterial hypertension	No
553	31-50	Male	No	No
566	31-50	Female	Obesity	No
570	18-30	Male	No	No
599	31-50	Female	No	No
710	31-50	Female	Systemic arterial hypertension	No
813	31-50	Male	No	No
837	31-50	Female	No	No
896	31-50	Female	Diabetes	December 2020
948	31-50	Female	No	No
1016	31-50	Female	No	No
1028	31-50	Female	Hypothyroidism - Thrombophilia	No
1043	31-50	Female	No	July 2020
1062	18-30	Female	No	No
1109	31-50	Female	No	No
1135	31-50	Female	No	No
1167	31-50	Male	No	No
1217	31-50	Male	No	No
1337	18-30	Male	No	No
1354	31-50	Female	No	No
1377	31-50	Male	No	No
1488	31-50	Female	No	No
1506	18-30	Male	No	No
1554	18-30	Female	No	No
1575	31-50	Female	No	December 2020
1588	18-30	Female	No	No
1617	31-50	Female	No	No

Supplementary Table 1: Detailed cohort data. Complete information for each individual included in the study.
