# Supplementary material for: Booster dose of BNT162b2 after two doses of CoronaVac improves neutralization of SARS-CoV-2 Omicron variant
Source: Commun Med (Lond). 2022 Jun 29;2:76. doi: 10.1038/s43856-022-00141-4 (PMC9242982; doi:10.1038/s43856-022-00141-4)
Supplement: Supplementary file 3 — Description of Additional Supplementary Files [file 43856_2022_141_MOESM3_ESM.pdf]

Description of Additional Supplementary Files

File Name: Supplementary Data

Description: VNT50 individual raw data
