# Supplementary material for: Booster dose of BNT162b2 after two doses of CoronaVac improves neutralization of SARS-CoV-2 Omicron variant
Source: Commun Med (Lond). 2022 Jun 29;2:76. doi: 10.1038/s43856-022-00141-4 (PMC9242982; doi:10.1038/s43856-022-00141-4)
Supplement: Supplementary file 4 — Reporting Summary [file 43856_2022_141_MOESM4_ESM.pdf]

nature research

Corresponding author(s): Mauricio L. Nogueira

Last updated by author(s): Jun 10, 2022

Reporting Summary

Nature Research wishes to improve the reproducibility of the work that we publish. This form provides structure for consistency and transparency in reporting. For further information on Nature Research policies, see our Editorial Policies and the Editorial Policy Checklist.

Statistics

For all statistical analyses, confirm that the following items are present in the figure legend, table legend, main text, or Methods section.

n/a	Confirmed	
☐	☒	The exact sample size (n) for each experimental group/condition, given as a discrete number and unit of measurement
☐	☒	A statement on whether measurements were taken from distinct samples or whether the same sample was measured repeatedly
☐	☒	The statistical test(s) used AND whether they are one- or two-sided<br>*Only common tests should be described solely by name; describe more complex techniques in the Methods section.*
☒	☐	A description of all covariates tested
☐	☒	A description of any assumptions or corrections, such as tests of normality and adjustment for multiple comparisons
☐	☒	A full description of the statistical parameters including central tendency (e.g. means) or other basic estimates (e.g. regression coefficient) AND variation (e.g. standard deviation) or associated estimates of uncertainty (e.g. confidence intervals)
☐	☒	For null hypothesis testing, the test statistic (e.g. F, t, r) with confidence intervals, effect sizes, degrees of freedom and P value noted<br>*Give P values as exact values whenever suitable.*
☒	☐	For Bayesian analysis, information on the choice of priors and Markov chain Monte Carlo settings
☒	☐	For hierarchical and complex designs, identification of the appropriate level for tests and full reporting of outcomes
☒	☐	Estimates of effect sizes (e.g. Cohen's d, Pearson's r), indicating how they were calculated

Our web collection on statistics for biologists contains articles on many of the points above.

Software and code

Policy information about availability of computer code

Data collection	No software was used to collect the data.
Data analysis	For data analysis, GraphPad Prism 6 was used.

For manuscripts utilizing custom algorithms or software that are central to the research but not yet described in published literature, software must be made available to editors and reviewers. We strongly encourage code deposition in a community repository (e.g. GitHub). See the Nature Research guidelines for submitting code & software for further information.

Data

Policy information about availability of data

All manuscripts must include a data availability statement. This statement should provide the following information, where applicable:
- Accession codes, unique identifiers, or web links for publicly available datasets
- A list of figures that have associated raw data
- A description of any restrictions on data availability

All source data included in the study is well described and available as Supplementary Data

Field-specific reporting

Life sciences study design

All studies must disclose on these points even when the disclosure is negative.

Sample size	The total sample size was determined in a way that all time-points evaluated could have the same number of samples. Thus, 90 samples were included in the study, distributed in 3 collection time-points (30 per time-point).
Data exclusions	No data was excluded from the analysis.
Replication	All attempts at replication of the experiments were successful.
Randomization	Allocation of the samples in each group occurred in a randomized way, in order to avoid any bias in the group analysis.
Blinding	From a larger amount of samples, the selection of samples that would compose each of the time-point groups was blinded and totally randomized.

Reporting for specific materials, systems and methods

We require information from authors about some types of materials, experimental systems and methods used in many studies. Here, indicate whether each material, system or method listed is relevant to your study. If you are not sure if a list item applies to your research, read the appropriate section before selecting a response.

Materials & experimental systems

n/a	Involved in the study	
☒	☐	Antibodies
☐	☒	Eukaryotic cell lines
☒	☐	Palaeontology and archaeology
☒	☐	Animals and other organisms
☐	☒	Human research participants
☒	☐	Clinical data
☒	☐	Dual use research of concern

Methods

n/a	Involved in the study	
☒	☐	ChIP-seq
☒	☐	Flow cytometry
☒	☐	MRI-based neuroimaging

Eukaryotic cell lines

Policy information about cell lines

Cell line source(s)	Vero cell line (ATCC CCL-81)
Authentication	Cells were authenticated by ATCC
Mycoplasma contamination	Cell lines tested negative to mycoplasma contamination.
Commonly misidentified lines (See ICLAC register)	No misidentified cell lines were used in this study

Human research participants

Policy information about studies involving human research participants

Population characteristics	All individuals included in the study were under the CoronaVac vaccination scheme against COVID-19. As explained above, the selection of the participants occurred in a randomized way, and was not considered relevant, for the conduction of the analysis, the selection by demographical data such as gender or age.
Recruitment	The participants are volunteers in a bigger study to evaluate the effectiveness and safety of the CoronaVac vaccination protocol, with the BNT162b2 booster dose. From this bigger study, a subgroup of samples, from each collection time-point evaluated, were randomly selected for our in vitro analysis here presented.
Ethics oversight	Oswaldo Cruz Foundation (42898621.9.0000.5091)

Note that full information on the approval of the study protocol must also be provided in the manuscript.
